# Swedish traveller with *Plasmodium knowlesi *malaria after visiting Malaysian Borneo

**DOI:** 10.1186/1475-2875-8-15

**Published:** 2009-01-16

**Authors:** Ulf Bronner, Paul CS Divis, Anna Färnert, Balbir Singh

**Affiliations:** 1 Unit of Infectious Diseases, Department of Medicine, Karolinska Institute, Karolinska University Hospital Solna, Stockholm, Sweden; 2 Malaria Research Centre, Faculty of Medicine & Health Sciences, University Malaysia Sarawak, 93150 Kuching, Sarawak, Malaysia

## Abstract

*Plasmodium knowlesi *is typically found in nature in macaques and has recently been recognized as the fifth species of *Plasmodium *causing malaria in human populations in south-east Asia. A case of knowlesi malaria is described in a Swedish man, who became ill after returning from a short visit to Malaysian Borneo in October 2006. His *P. knowlesi *infection was not detected using a rapid diagnostic test for malaria, but was confirmed by PCR and molecular characterization. He responded rapidly to treatment with mefloquine. Evaluation of rapid diagnostic kits with further samples from knowlesi malaria patients are necessary, since early identification and appropriate anti-malarial treatment of suspected cases are essential due to the rapid growth and potentially life-threatening nature of *P. knowlesi*. Physicians should be aware that knowlesi infection is an important differential diagnosis in febrile travellers, with a recent travel history to forested areas in south-east Asia, including short-term travellers who tested negative with rapid diagnostic tests.

## Background

*Plasmodium knowlesi *occurs in nature in long-tailed and pig-tailed macaques (*Macaca fascicularis *and *Macaca nemestrina*, respectively), that are commonly found in forested areas of south-east Asia [1]. Until a few years ago, infection with *P. knowlesi *was regarded as a rare disease, occurring only sporadically in humans [2]. The recent findings of a large number of infected patients in Malaysia [3-5], especially in Sarawak, Malaysian Borneo and other reports of human cases acquired in Thailand [6], Myanmar [7], the Philippines [8] and Singapore [9], have changed the perception of *P. knowlesi*. This parasite is clearly the fifth species of *Plasmodium*, causing malaria in humans in south-east Asia [10]. It has the shortest erythrocytic cycle among the primate malarias, only 24 hours [1], and high parasitaemias with fatal outcome in humans can occur [4]. Under the microscope, it is not possible to distinguish between *P. knowlesi *and *P. malariae *due to their similar morphological characteristics [1,3,10].

## Case presentation

In October 2006, a 35-year-old Swedish man presented to hospital in Stockholm with a two-day history of fever, sweats, headache and fatigue. He had a temperature of 40°C, but was otherwise in good general condition with a normal respiratory rate and blood pressure. Laboratory investigations revealed normal haemoglobin concentration (154 g/L), leucopaenia (2.2 × 10^9^/L), moderate thrombocytopenia (58 × 10^9^/L) and an elevated C-reactive protein concentration of 109 mg/L (normal < 10 mg/L). Plasma creatinine was normal, while plasma bilirubin (31 μmol/L) was just above the upper limit of the normal range and alanine transaminase (1.68 μcat/L) was slightly increased (normal < 1.20 μcat/L).

**Figure 1:**
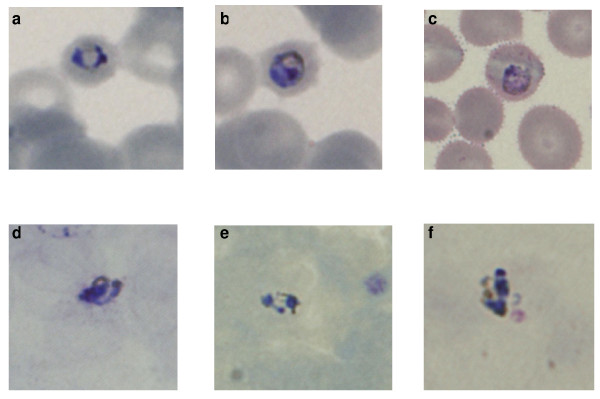
**Malaria parasites observed in blood films from the patient**. Photos 'a' to 'c' and 'd' to 'f' are of thin and thick blood films, respectively. All the photos are from Giemsa-stained blood films except for photos 'b' and 'c' that are from a film stained with Field's stain.

The patient had recently returned from a two-week holiday in Sarawak, Malaysian Borneo. He had trekked in the jungle of the Bario Highlands during the last seven days of his holiday, at altitudes between 800 and 1,400 m. He had not taken any anti-malarial chemoprophylaxis. His symptoms started 11 days after leaving the Bario Highlands. There was a strong suspicion of malaria, and although a rapid diagnostic test (Now^® ^Malaria Test, Binax Inc., Maine, USA) was negative, oral mefloquine therapy was started while waiting for the results of the blood film examination. Malaria parasites were detected by microscopy in 0.1% of erythrocytes. The *Plasmodium *species could not be identified conclusively, but *Plasmodium malariae *was suspected, since late trophozoites were observed and the infected red blood cells were not enlarged (Figure 1). The patient improved rapidly following mefloquine treatment and was discharged afebrile after two days. The lowest recorded haemoglobin and platelet levels were 95 g/L and 34 × 10^9^/L, respectively. Two blood cultures were negative and a pulmonary x-ray was normal.

**Figure 2:**
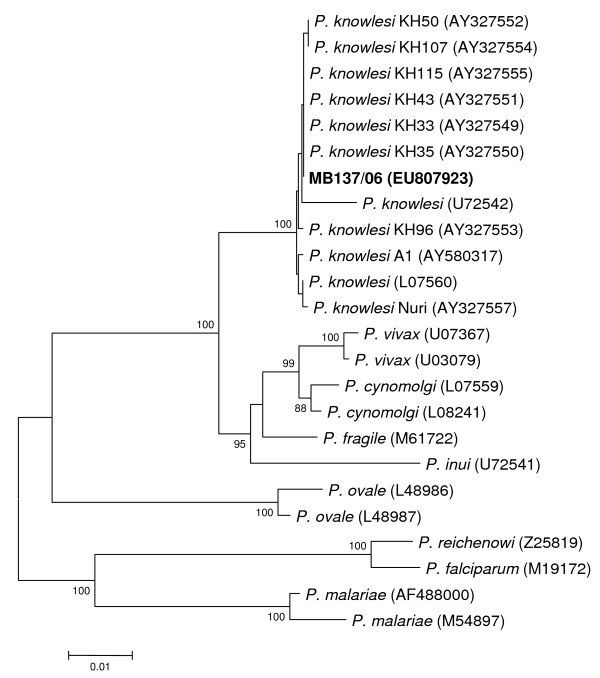
**Phylogenetic tree based on the A-type small subunit ribosomal RNA gene sequences of *Plasmodium *species**. The tree was constructed according to the neighbour-joining method with 1,000 replicates and only bootstrap values above 80% are shown. Newly generated nucleotide sequence for the sample from the Swedish patient (MB137/06) is in bold. GenBank accession numbers are in brackets.

Due to the uncertain identification by microscopy, PCR assays for *P. falciparum, Plasmodium vivax, Plasmodium ovale *and *P. malariae *were performed in Sweden. These assays were negative, but microscopy results that were suggestive of *P. malariae *and the patient's travel history led to the suspicion of a *P. knowlesi *infection. A frozen blood sample, collected when the patient was admitted to hospital, was sent to the Malaria Research Centre at University Malaysia Sarawak for further analysis. The infection was identified as a single *P. knowlesi *infection following DNA extraction and examination by PCR assays for *P. falciparum, P. vivax, P. malariae, P. ovale *and *P. knowlesi *as described previously [3]. PCR amplification, cloning and sequencing of the A-type small subunit ribosomal (SSU r) RNA gene was also undertaken, followed by phylogenetic analysis as described earlier [3]. The SSU rRNA gene sequence of the malaria parasite from the Swedish patient was phylogenetically indistinguishable from those of other *P. knowlesi *isolates (Figure 2).

## Conclusion

The nested PCR assay and molecular characterization confirmed that the patient was infected with *P. knowlesi*. Since the incubation period of *P. knowlesi *in the liver is 9 to 12 days [2], this indicates that he most probably acquired his infection while jungle trekking in the Bario Highlands of Sarawak, Malaysian Borneo.

*Plasmodium knowlesi *parasites morphologically resemble those of *P. malariae *[1,2,10] and PCR assays can correctly differentiate between the two species [3]. However, PCR assays are not a rapid method of detection and are not a viable option for routine diagnosis. Rapid diagnostic tests are becoming an increasingly popular method for diagnosis of malaria [11], particularly by doctors on call and medical laboratory technologists in non-malaria endemic countries, who are not experienced in identifying malaria parasites by microscopy. It is possible that the low parasitaemia may have been a contributing factor to the inability of the rapid diagnostic test to detect knowlesi malaria in this Swedish patient, since these tests are not as sensitive as microscopy [12]. In a recent study of monoclonal antibodies against *Plasmodium *lactate dehydrogenase that have been developed for use in a rapid diagnostic test, it was shown that certain antibodies that were thought to be specific for *P. falciparum *and *P. vivax*, also bind to *P. knowlesi *[13]. However, it remains to be determined whether this, and commercially available rapid diagnostic tests, are able to detect *P. knowlesi *infections by examining a larger number of patient samples.

Early identification and adequate anti-malarial treatment of suspected cases are essential since *P. knowlesi *has a 24-hr erythrocytic cycle, and high parasitaemias with fatal outcome in humans can occur [4]. Human *P. knowlesi *infections resolve rapidly following treatment with chloroquine [3] and the current report indicates that mefloquine is also an effective anti-malarial for knowlesi malaria. However, in severely ill knowlesi malaria patients with high parasitaemias, management and treatment as for severe falciparum malaria has been recommended due to the rapid rate at which *P. knowlesi *can multiply [4].

Previous reports of human knowlesi malaria infections were of people that have either lived or worked in the forest and forest fringe areas of south-east Asia [10]. There has been a recent report of a Finnish traveller, who returned from Peninsular Malaysia with knowlesi malaria [14]. Taken together with this report of *P. knowlesi *infection in a Swedish traveller returning from Malaysian Borneo, this indicates that knowlesi malaria infections in travellers are probably not rare events, particularly since south-east Asia has become a popular tourist destination.

In conclusion, this report shows that *P. knowlesi *can infect short-term visitors to areas in south-east Asia, where knowlesi transmission occurs, and mefloquine was an effective anti-malarial. The *P. knowlesi *infection in the Swedish traveller was not detected by a rapid diagnostic test and evaluation of rapid diagnostic tests with further samples are necessary since early identification and appropriate anti-malarial treatment of suspected cases are essential, due to the rapid growth and potentially pathogenic nature of knowlesi malaria. Physicians should, therefore, be aware that knowlesi infection is an important differential diagnosis in febrile travellers with a recent travel history to forested areas in Southeast Asia, including short-term travellers, who tested negative with rapid diagnostic tests.

## Competing interests

The authors declare that they have no competing interests.

## Authors' contributions

UB, BS and AF wrote the paper, AF facilitated the study, UB was the physician responsible for the patient, BS supervised molecular characterization of parasites and analysis of sequence data, and PCSD was responsible for molecular detection, characterization and phylogenetic analysis of sequence data. All authors read and approved the final manuscript.

## Consent

Written informed consent was obtained from the patient for publication of this case report.

## References

[B1] Garnham PCC (1966). Malaria parasites and other haemosporidia.

[B2] Coatney RG, Collins WE, Warren McW, Contacos PG (1971). The primate malarias.

[B3] Singh B, Sung LK, Matusop A, Radhakrishnan A, Shamsul SSG, Cox-Singh J, Thomas AW, Conway D (2004). A large focus of naturally acquired *Plasmodium knowlesi *infections in human beings. Lancet.

[B4] Cox-Singh J, Davis TEM, Lee KS, Shamsul SSG, Matusop A, Ratnam S, Rahman HA, Conway DJ, Singh B (2008). *Plasmodium knowlesi *malaria in humans is widely distributed and potentially life threatening. Clin Infect Dis.

[B5] Vythilingam I, NoorAzian YM, Huat TC, Jiram AI, Yusri YM, Azahari AH, NorParina I, NoorRain A, LokmanHakim S (2008). *Plasmodium knowlesi *in humans, macaques and mosquitoes in peninsular Malaysia. Parasit Vectors.

[B6] Jongwutiwes S, Putaporntip C, Iwasaki T, Sata T, Kanbara H (2008). Naturally acquired *Plasmodium knowlesi *malaria in human, Thailand. Emerg Infect Dis.

[B7] Zhu HM, Li J, Zheng H (2006). Human natural infection of *Plasmodium knowlesi*. Chinese J Parasitol Parasit Dis.

[B8] Luchavez J, Espino F, Curameng P, Espina R, Bell, Chiodini P, Nolder D, Sutherland C, Lee K-S, Singh B (2008). Human infections with *Plasmodium knowlesi*, the Philippines. Emerg Infect Dis.

[B9] Ng OT, Ooi EE, Lee CC, Jarrod LP, Ng LC, Wong PS, Ming TT, Phang LJ, Sin LY (2008). Naturally acquired human *Plasmodium knowlesi *infection, Singapore. Emerg Infect Dis.

[B10] Cox-Singh J, Singh B (2008). Knowlesi malaria: newly emergent and of public health importance?. Trends Parasitol.

[B11] World Health Organization (2004). The use of malaria diagnostic tests.

[B12] Wongsrichanalai C, Barcus MJ, Muth S, Sutamihardja A, Wernsdorfer WH (2007). A review of malaria diagnostic tools: Microscopy and rapid diagnostic test (RDT). Am J Trop Med Hyg.

[B13] McCutchan TF, Piper RC, Makler MT (2008). Use of malaria rapid diagnostic test to identify *Plasmodium knowlesi *infection. Emerg Infect Dis.

[B14] Kantele A, Marti H, Felger I, Müller D, Jokiranta TS (2008). Monkey malaria in a European traveler returning from Malaysia. Emerg Infect Dis.

